# Bad Connection: Stent-to-Stent Fistulization After Common Bile Duct and Transjugular Intrahepatic Portosystemic Shunt Stenting

**DOI:** 10.14309/crj.0000000000001041

**Published:** 2023-05-06

**Authors:** Aaron G. Issac, Alix Youngblood, Chidi Enunwa, Hima Veeramachaneni, Anshika Khare, Mohammad Razvi, Francis Edward Levert

**Affiliations:** 1 Department of Medicine, Emory University School of Medicine, Atlanta, GA; 2 Division of Digestive Diseases, Emory University School of Medicine, Atlanta, GA

**Keywords:** Common bile duct fistula, Transjugular intrahepatic portosystemic shunt fistula, Bare metal TIPS stent

## Abstract

A 29-year-old man with chronic portal venous thrombosis resulting in portal biliopathy required stenting of his common bile duct (CBD) and underwent a transjugular intrahepatic portosystemic shunt (TIPS) procedure to decrease portal pressures. He later presented with abdominal pain in the setting of prolonged CBD stent placement and was found to have air within his TIPS stent with a fistula on endoscopic retrograde cholangiopancreatography between his fully covered CBD stent and bare metal TIPS stent. There was concern that further intervention would lead to an air embolus. We suggest that when multiple stents are indicated, stent selection with close monitoring is critical.

## INTRODUCTION

Portal biliopathy refers to intrahepatic and extrahepatic biliary tree abnormalities, which occur in patients with portal cavernoma due to extrahepatic portal vein obstruction.^1^ Chronic engorgement of venous collaterals is theorized to cause ischemia and biliary damage through prolonged direct pressure and insufficient blood supply. Portal biliopathy is a progressive condition although most patients remain asymptomatic. There is little data on what factors affect the occurrence of symptomatic portal biliopathy, such as extent of initial thrombosis or underlying etiology of portal hypertension. Patients with symptomatic disease (5%–30%) have very advanced cholangiographic abnormalities including ectasia, angulation, strictures, and aneurysmal dilations, which result in recurrent episodes of biliary pain, cholangitis, and obstruction.^2^

## CASE REPORT

**Figure 1.**
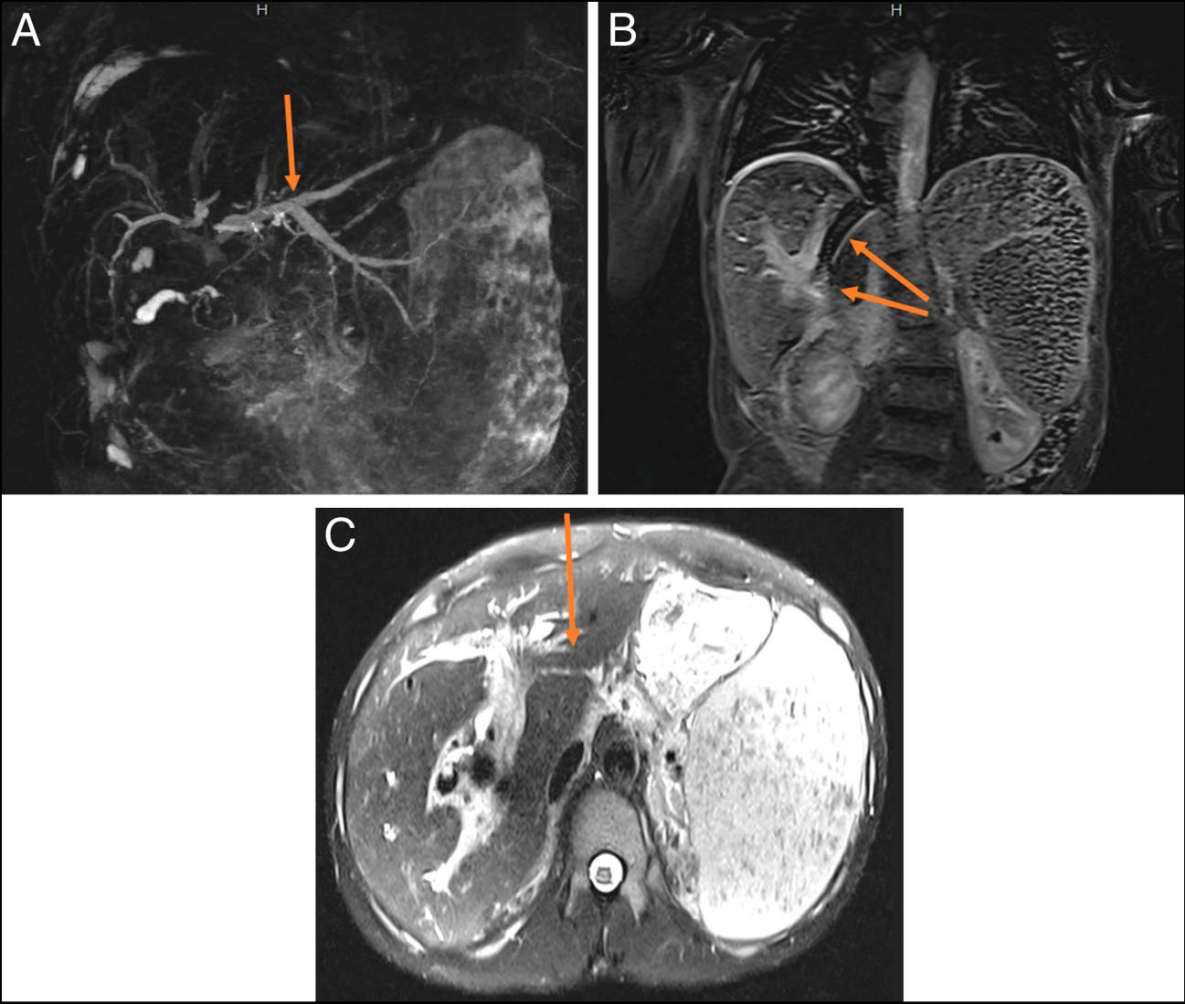
Magnetic resonance imaging of the abdomen and pelvis (T2 haste) showing a column of air within the TIPS stent and fistulous connection between the TIPS and CBD stents in different views: (**a**) intrahepatic biliary ductal dilation (MRCP 3D reconstruction), (**b**) the TIPS stent with intraluminal air (upper arrow) and concern for fistulous connection with the CBD stent (lower arrow) (axial view), and (**c**) the TIPS stent with intraluminal air (coronal view). CBD, common bile duct; MRCP, magnetic resonance cholangiopancreatography; TIPS, transjugular intrahepatic portosystemic shunt.

The patient was a 29-year-old man who initially presented to our center with concerns of generalized body aches and was found to have a column of air within his transjugular intrahepatic portosystemic shunt (TIPS) stent and a fistulous connection between his common bile duct (CBD) plastic stent and uncovered metal TIPS stent (Figure 1). He had a history of chronic portal venous thrombosis in the setting of a hypercoagulable state from systemic lupus erythematous with a positive lupus anticoagulant. This led to cavernous transformation resulting in portal biliopathy with complications of recurrent ascending cholangitis, bacteremia, and abscess formation. This was managed with multiple CBD stents and eventually a TIPS procedure with an uncovered stent at an outside center.

**Figure 2.**
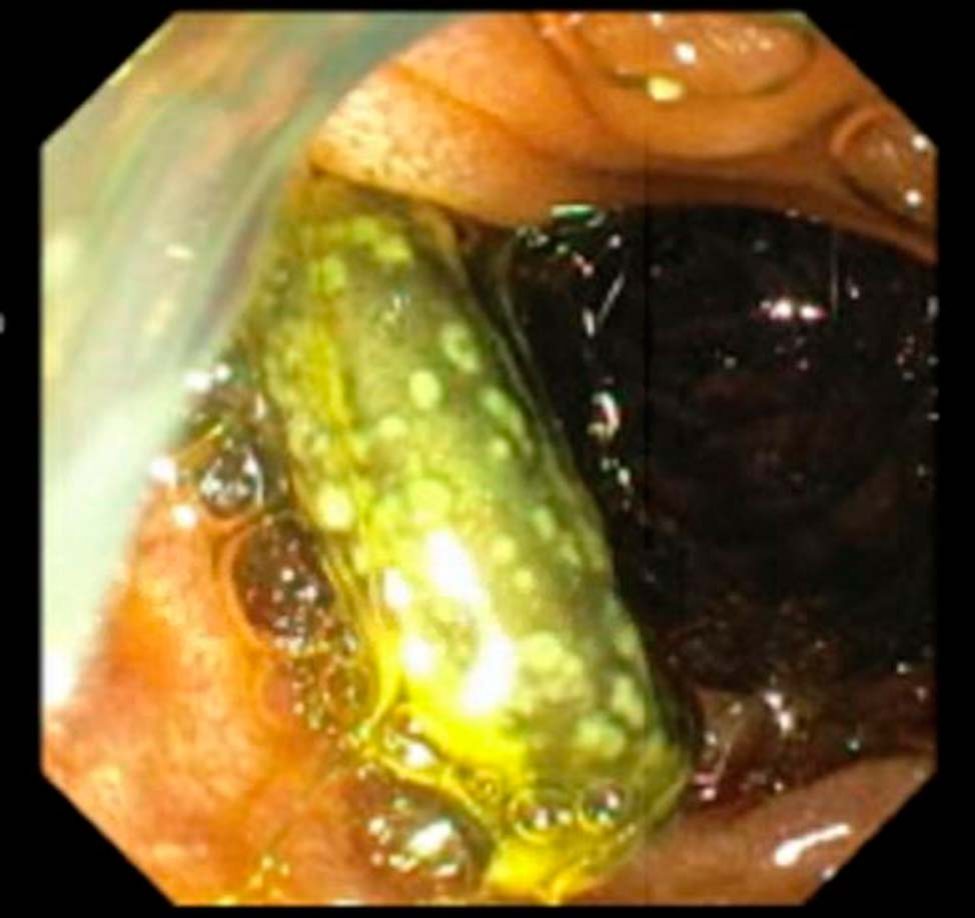
Endoscopic retrograde cholangiopancreatography showing an occluded stent at the major duodenal papilla.

For unclear reasons, the plastic CBD stent was not replaced for over a year and the patient had declined prior surgical interventions such as a mesocaval shunt. When he presented to our center, the patient had abdominal pain that was worked up by CT, which showed a column of air within a TIPS stent that was occluded at both the proximal and distal ends with concern for a fistula between the CBD stent and the TIPS stent. The gastroenterology team further evaluated this by endoscopic retrograde cholangiopancreatography (Figure 2), during which time, the CBD stent was removed; a fistulous connection between the prior CBD stent and the uncovered TIPS stent was confirmed; and a covered metal stent was placed in the CBD. The patient also had a new Citrobacter braakii bacteremia during this hospitalization, which further confirmed that the GI enteric flora was able to enter the systemic circulation through the fistulous connection. Further intervention by interventional radiology was impeded by concern that the column of air within the TIPS stent would lead to an air embolus during any procedure. The patient was further evaluated by general surgery and vascular surgery; however, further definitive treatment was deferred in the setting of severe thrombocytopenia secondary to immune-mediated thrombocytopenia. His immune-mediated thrombocytopenia was managed with medical therapy initially but was refractory requiring splenectomy with initial improvement, although later it relapsed. He was discharged with close follow-up with planned outpatient CBD stent exchange.

## DISCUSSION

No clear guidelines exist for the management of symptomatic cases of portal biliopathy, although in prior cases, a stepwise approach is often used depending on the presumed etiology. Initial management, if CBD calculi or cholangitis is suspected, is with endoscopic retrograde cholangiopancreatography, balloon sweep, and stenting. Although difficult to treat, calculi may result in more advanced endoscopic techniques such as large balloon sphincterotomy and intraductal lithotripsy.^2^ Initial biliary tree stent management is centered around plastic stenting or even self-expanding metal stents. It is important to note that metal stents can be difficult to retrieve and can complicate subsequent procedures. For biliary strictures without complicating cholangitis or shunt contraindications, most centers may use surgical portosystemic shunt procedures such as TIPS as first-line management. Other indications include persistent symptoms refractory to other therapies. TIPS is associated with significant clinical improvement, with only one-third of patients continuing to have symptoms afterward.^3^

The first TIPS procedure with a metal stent was performed on humans in 1988 for end-stage portal hypertension.^4^ The procedure has since gained notoriety, with the most common indications including refractory esophageal varices and ascites. TIPS is designed to decrease portal pressure by placing a stent between the portal and hepatic veins; thus, it can be considered in other causes of portal hypertension such as portal biliopathy.^5^ Initial stent selection during the early years of TIPS was limited to bare metal stents. During the early 2000s, the introduction of covered metal stents was trialed under direct comparison with bare metal stents showing higher rates of 4 and 5-year post-TIPS survival and lower rates of gastrointestinal bleeding and refractory ascites.^6^ The cumulative restenosis rate is significantly lower, and importantly, the overall rate of secondary interventional therapy is lower (20.6% for covered stents vs 49.6% for bare metal stents).^7^ With an improved safety profile, covered stents remain the first-line choice for TIPS procedures.^8^

The only note of biliary TIPS fistulous complications with TIPS stents are those associated with occlusion or stenosis of the TIPS stent leading to TIPS-associated biliary fistula formation.^9^ CBD stents are not associated with fistulous connections as a complication, with only an isolated report of choledochoduodenal fistula after significant migration of a bare metal stent.^10^ A TIPS stent-to-CBD stent fistulous connection has not been described in the literature. Bare metal stents have an increased likelihood of creating a fistulous connection with other stents because of their design, compared with covered metal stents. Covered metal stents have been increasingly used for TIPS shunts because of the decreased number of complications associated with them and should especially be considered in patients with portal biliopathy because they often require multiple interventions and CBD stents. As demonstrated in this case, when concomitant procedures such as common bile duct stenting are required, selection of the stent type is critical to reduce complication including stent-to-stent fistulization.

## DISCLOSURES

Author contributions: All authors reviewed and approved the final manuscript. H. Veeramachaneni, A. Khare, M. Razvi, and FE Levert: article concept and design and revision of the manuscript. AG Issac and A. Youngblood: acquisition of data and drafting of the manuscript. C. Enunwa: revision of the manuscript. AG Issac is the article guarantor.

Financial disclosure: None to report.

Informed consent was obtained for this case report.
